# GRADUATING MEDICAL STUDENTS’ PERFORMANCE ON A GERIATRICS KNOWLEDGE TEST IS ASSOCIATED WITH ATTITUDES

**DOI:** 10.1093/geroni/igae098.0562

**Published:** 2024-12-31

**Authors:** Apoorva Rangan, Yochai Shavit, Deborah Kado

**Affiliations:** Stanford University, Stanford, California, United States; Stanford University, Stanford, California, United States; Stanford University, Stanford, California, United States

## Abstract

Older adults are overrepresented among patients, with half of outpatient visits and 40% of hospitalizations for adults over 65. Training physicians in age-friendly care is imperative. Yet, medical school graduates’ knowledge of core geriatrics competencies is unknown, as are factors associated with students’ knowledge base. 30 of the 82 graduating students at the Stanford School of Medicine completed a three-part survey. Geriatrics knowledge was assessed using a validated 18-item UCLA Geriatrics Knowledge Test (GKT) and ten questions from the Geriatrics Review Syllabus (GRS). Attitudes towards older patients were assessed using the validated 14-item UCLA Geriatrics Attitudes Assessment. Finally, students reported their curricular exposure to geriatrics and career interests. The Stanford Institutional Review Board waived institutional review. Two researchers independently analyzed results in R. Geriatrics exposure was limited; 1 student took a preclinical elective and 2 took clinical electives. Most students incorrectly completed at least half of the knowledge assessment. All students missed at least 7/28 questions overall (M = 8.0, SD = 6.7), with students performing better on the GKT (M = 8.4, SD = 5.1) than the GRS portion (M = -0.4, SD = 3.0). Most students had neutral or slightly positive attitudes towards older adults (M = 3.58, SD = 0.87). Attitude scores were moderately associated with improved performance on the knowledge assessment (r = 0.55, p < 0.0001). In conclusion, graduating students did not meet minimum geriatrics competencies measured by a knowledge assessment. Attitudes towards older adults were associated with performance, supporting future intersectional interventions in ageism.

